# Depression and associated factors among primary school adolescents with hearing impairment in Kampala district, Uganda: A cross-sectional study

**DOI:** 10.1371/journal.pmen.0000588

**Published:** 2026-06-18

**Authors:** Mark Andrew Muyanga, Justine Diana Namuli, Joyce Sserunjogi Nalugya, Mark Kaddumukasa, Martha Sajatovic, Emmanuel Kiiza Mwesiga, Sadat Katama, Dickens Akena

**Affiliations:** 1 Department of Psychiatry, School of Medicine, College of Health Sciences, Makerere University, Kampala, Uganda; 2 Department of Psychiatry, Mulago National Referral Teaching Hospital, Kampala, Uganda; 3 Department of Medicine, Makerere University College of Health Sciences, Kampala,‌‌ Uganda; 4 Neurological and Behavioral Outcomes Center, University Hospitals Cleveland Medical Center,‌‌ Cleveland, Ohio, United States of America; Baylor Scott and White Research Institute, UNITED STATES OF AMERICA

## Abstract

A large number of adolescents with hearing impairment experience socio-emotional and structural challenges that may put them at risk for depression. However, depression is often undiagnosed among them due to lack of appropriate, culturally relevant tools for its assessment. This study set out to determine the prevalence of depression and its associated factors in this vulnerable population. A cross-sectional study was conducted among adolescents aged 10–17 years with hearing impairment, attending three primary schools of the deaf across Kampala City in Uganda. Depression was assessed using the Mini International Neuropsychiatric Interview for children and adolescents (MINI-KID), which underwent linguistic adaptation and translation into Ugandan Sign Language through a collaborative process involving sign language teachers and students, and administered in video format to ensure clarity and inclusion. Data were analyzed with Stata version 17. Modified Poisson regression with robust standard errors was used to examine associations. A total of 116 adolescents participated (mean age = 14.5 ± 1.9 years; 51.7% female). The lifetime prevalence of depression was 60.3%, with point prevalence at 34.5%. Depression was more frequent among females, though these differences were not statistically significant. Living in father-only households was associated with higher likelihood of depression (IRR = 2.11, 95% CI: 1.60–2.77 p < 0.001), while not experiencing or witnessing excessive disciplinary measures at school was associated with a lower likelihood of depression (IRR = 0.65, 95% CI: 0.49–0.87, p = 0.003). Using a linguistically adapted version of the MINI-KID, we found that depression is highly prevalent among adolescents with hearing impairment in Kampala. Strengthening family support, improving access to sign language-trained professionals, and implementing inclusive school-based interventions are key to identifying and managing depression in this population.

## Introduction

Hearing impairment (HI) is one of the most prevalent forms of disability, affecting over 1.5 billion people globally, with over 430 million of them having disabling HI. Projections indicate that HI is expected to rise to 2.5 billion by 2050 with the greatest burden in low- and middle- income countries where access to early intervention and specialized care remains limited [1]. Uganda alone is estimated to have about 1.65 million people living with HI [2], more than half of whom are below the age of 18 [3].

The experience of HI can engender a host of difficulties, including communication bar, i.e.,rs, social isolation, and limited access to educational and employment opportunities [4–6]. These challenges can considerably impede an adolescent’s overall well-being and heighten their vulnerability to mental health issues. One of the commonest mental health challenges among adolescents with HI includes depression [7]. Literature shows a high prevalence of depression among adolescents with HI, including findings from a study in South Kenya which reported a prevalence of 54.7% [8]. Beyond elevated prevalence, studies show important adverse consequences of depression among adolescents with HI including reduced health-related quality of life [9], school disengagement and suicidality [10], and bullying which is common and linked to negative outcomes [11]. These consequences mirror those observed in other chronic conditions such as HIV, where depression compounds already existing social and health burdens [12].

Despite the high prevalence of depression and its significant impact among persons with HI, little research has been done in this area. One of the main barriers to the screening and diagnosis of depression among persons with HI in Africa is the lack of standardized tools. Whereas there are self-administered instruments for the assessment of depression like the patient health questionnaire (PHQ) [13,14], low levels of functional and mental health literacy pose significant barriers to their routine use in clinic and community settings. Furthermore, these tools were not designed for persons with HI; they rely heavily on verbal communication yet people with HI rely mostly on visual communication [15]. In addition, most commonly used depression screening and diagnostic tools have not been translated or culturally adapted into sign languages in diverse settings [16]. Use of such tools in written language or spontaneously translating them into sign language could result in misdiagnosis or miscommunication [16]. To our knowledge, there is currently no standardized depression assessment tool available in Ugandan Sign Language, which limits the ability of clinicians and researchers to accurately identify depression among adolescents with HI. This study aimed to determine the prevalence of depression and associated factors among school going adolescents with HI in Kampala district, Uganda.

## Methods

### Ethical statement

Ethical approval for this study was obtained from the Makerere University School of Medicine Research and Ethics Committee (Mak-SOMREC) on 15^th^/07/2024, under approval number Mak-SOMREC-2024–902, prior to starting data collection. Written informed consent was obtained from the caregivers or legal guardians of all participating learners, and written assent was obtained from the learners themselves. Sign language interpreters also provided consent to participate in the communication facilitation process. Any participant found to have depression or suicidal ideation was referred to the Child and Adolescent Psychiatry Clinic at Mulago National Referral Hospital for further evaluation and management. To protect confidentiality, all identifying information was removed during data entry and each questionnaire was assigned a unique serial code. No personal identifiers were retained in the final dataset.

### Study design and setting

This was a cross-sectional study design conducted in three schools of the deaf located in Kampala district, over a six-week period from the 5th of October 2024–16^th^ November 2024. These were; Mulago School of the Deaf, Uganda School for the Deaf and Uganda Society for Deaf Vocational Training Centre; which had a total population of 418 pupils during the study period. All data collection was carried out strictly over the weekends; on Saturdays and Sundays so as to ensure that the academic routines of the students were not disrupted.

### Translation of the MINI-KID into sign language

To ensure cultural and linguistic appropriateness, the Depression Module of the MINI-KID was translated into Ugandan Sign Language (USL). The translation followed a committee-based approach in which a team of sign language teachers and adolescents with HI worked together to review and adapt each item so that the intended meaning of the questions was retained while making them understandable within the structure of USL.

The process involved a translation team comprising two adolescents with HI and three sign language teachers who were familiar with the communication needs of adolescents with HI, to ensure that translations made were age and context appropriate. Together they reviewed all MINI-KID items to identify equivalent USL signs and resolve expressions without direct sign equivalents while maintaining the intent and diagnostic meaning of each question. Where a direct sign equivalent was not available, the team discussed alternative ways of expressing the concept in USL so that it could be clearly understood by the study participants.

Each item and response was subsequently video-recorded, with teachers signing the questions in USL. A third teacher independently reviewed the recordings for accuracy and the final version was uniformly used for all participants during data collection. At assessment, the video was projected to each participant who each had response sheets requiring circling either a “yes” or “no” to each projected question. Sign language teachers acting as trained research assistants were available to give and clarify instructions.

This approach ensured that the tool was linguistically accessible, culturally relevant and standardized for use among the participants. Given that there are currently no standardized assessment tools available in USL, this translation process was undertaken to improve access to structured mental health assessment for adolescents with HI who communicate using USL. The intention of this process was to improve the linguistic accessibility and cultural relevance of the MINI-KID for adolescents using USL and should therefore be understood as a linguistic and contextual adaptation rather than a formal psychometric validation of the depression module of the MINI-KID. Access details for the adapted USL MINI-KID video materials are provided as S2 Text.

### Sampling procedure

A sample size of 116 participants was calculated using OpenEpi version 2.3.1, based on an estimated prevalence rate of 10% [17,18].

The schools were purposively selected due to their relatively larger student populations which would enable achievement of the estimated sample size, as well as their status as government aided schools which enabled ease of obtaining administrative approval and access. Proportionate sampling was used to determine how many participants came from each school and simple random sampling was thereafter employed to determine the final participants.

### Inclusion and exclusion criteria

The study included adolescents with confirmed hearing impairment, aged 10–17 years, attending one of the selected schools of the deaf. We excluded adolescents with known diagnoses of schizophrenia, bipolar disorder, or having co-existing severe physical disability such as profound visual impairment, that would make participation in assessment difficult or interfere with completion of the study procedures. This exclusion criterion did not refer to ordinary self-reported physical conditions which were captured separately as a study variable among eligible participants. Those who were eligible for the study assented for the study while their parents/guardians provided written informed consent.

### Study instruments

Depression of the respondents was diagnosed using the depression module of the Mini International Neuropsychiatric Interview for Children and Adolescents (MINI-KID). The MINI-KID [19] is a structured diagnostic interview widely employed to evaluate various psychiatric disorders in children and adolescents aged 6–17, making it suitable for assessing depression among adolescents. Although it has not undergone formal psychometric validation in Uganda, it has demonstrated acceptable reliability and validity in several studies conducted in African settings and has been used in previous research involving children and adolescents in Uganda. It encompasses a comprehensive assessment of psychiatric conditions, including mood disorders, such as depression. The interview involves a series of standardized questions and diagnostic criteria, facilitating a reliable and valid diagnosis of depression in this age group.

A socio-demographic standard questionnaire was used to record the socio-demographic characteristics including; age, sex, grade, what age one started school, at what age one knew about one’s hearing loss, what caused the hearing loss, and the various factors relating to the health system, family, school, one’s community or the individual him or herself, that could have contributed to that individual’s mental state at that time or before. The socio-demographic questionnaire used to collect participant characteristics is provided as S1 Text.

### Data management and analysis

The data was entered in Microsoft Excel 2016 and analysed using the STATA version 17 analysis tool. Data was checked for completeness and cleaned before analysis. Summary statistics were done and numerical data summarized as mean and standard deviation. The primary outcome variable for statistical analysis was lifetime depression, defined as the presence of either a current depressive episode or a previous depressive episode as determined using the depression module of the MINI-KID. Point prevalence of depression was calculated separately and reported descriptively to indicate the proportion of adolescents experiencing an episode of depression at the time of assessment. Multivariable Poisson regression with robust standard errors was employed to identify factors significantly associated with dependent variable based on the bivariate analysis conducted using the Chi-square test or Fischer’s exact test. Variables with p-values of less than 0.05 in the bivariate analysis together with variables considered theoretically relevant based on existing literature, were included in the multivariable Poisson regression model. Multicollinearity among the independent variables was assessed using the variance inflation factor (VIF). A temporary linear model including the same predictors was fitted to estimate VIF values. The mean VIF was 1.14, indicating no evidence of problematic multicollinearity.

## Results

Among the 116 enrolled adolescents with HI, the majority (68.1%) were between 14 and 17 years of age, with a mean age of 14.5 years. Slightly more than half were female. Most of the participants (80.2%) reported being born with HI (See Table 1).

**Table 1:** Socio-demographic characteristics of school-going adolescents with hearing impairment in Kampala District (N = 116).

Variable	Categories	n (%)
Age	14.5(1.9)	
Age group (years)	10–13	37(31.9)
	14–17	79(68.1)
Sex	Male	56(48.3)
	Female	60(51.7)
Grade	Lower Primary (P.3 & P.4)	19(16.3)
	Upper Primary (P.5, P.6 & P.7)	97(83.7)
Age of starting school	5 years and above	53(46.5)
	Above 5 years	61(53.5)
Age of knowing about your hearing loss	5 years and above	8(6.9)
	Before 5 years	15(12.9)
	I was born with HI	93(80.2)
Reason for loss of hearing	Don’t know	92(79.3)
	Malaria	15(12.9)
	Other Causes (drugs like quinine, noise related, others)	9(7.8)

**Fig 1:**
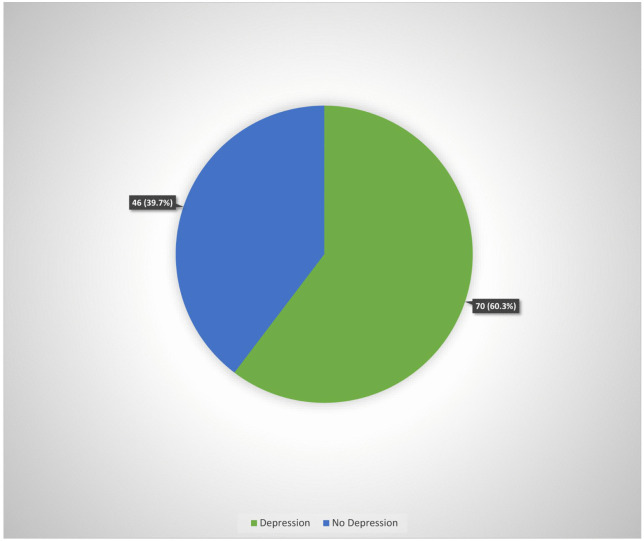
Lifetime prevalence of depression (N = 116). 60.3% of all the respondents had experienced depression at some point in their life.

**Fig 2:**
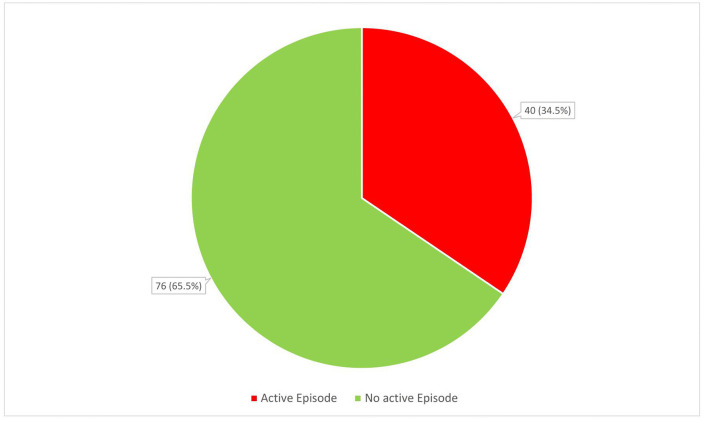
Point prevalence of depression (N = 116). The point prevalence of depression was 34.5%, indicating that over one-third of participants were experiencing either a current or recurrent depressive episode during the assessment period.

The point prevalence of depression was 34.5% and, 60.3% reported that they had experienced depression at some point in their lives (see Fig 1 and Fig 2). Suicidal ideation was reported by almost one in five of the study participants (21%).

### Factors associated with depression

Age and gender were not significantly associated with depression as their p values were beyond 0.05, (p = 0.135 and p = 0.289, respectively) (See Table 2) However, who the adolescent stays with (living arrangements) was found to be significantly associated with depression (**p** = **0.028**). Witnessing excessive disciplinary measures was also significantly associated with depression (**p** = **0.044**), with 17.1% of those exposed reporting depression compared to 4.3% of those who had not (See Table 2).

**Table 2:** Bivariate analysis for factors associated with depression among school-going adolescents with hearing impairment (N = 116).

Characteristic	Depression (N = 70) n (%)	No depression (N = 46) n (%)	P value
**Age group**			
10-13 years	26(37.1)	11(23.9)	
14-17 years	44(62.9)	35(76.1)	0.135
**Gender**			
Male	56(48.3)	25(54.4)	
Female	60(51.7)	21(45.6)	0.289
**At what age did you know about your HI**			
I was born with HI	57(81.4)	36(78.3)	
Before 5 years	9(12.9)	6(13.0)	
5 years and above	4(5.7)	4(8.7)	0.825 ^*i*^
**Who do you stay with?**			
Both parents	31(44.3)	31(67.4)	
Mother only	25(35.7)	11(23.9)	
Father only	7(10.0)	0(0)	
Others	7(10.0)	4(8.7)	**0.028** ^***i***^
**Do you use a hearing aid?**			
No	52(74.3)	40(87.0)	
Yes	18(25.7)	6(13.0)	0.099
**Have you experienced or witnessed excessive disciplinary measures at school?**			
Yes	12(17.1)	2(4.3)	
No	58(82.9)	44(95.6)	**0.044** ^***i***^
**Do you have any self-reported physical condition?**			
Yes	24(34.8)	18(39.1)	
No	45(65.2)	28(60.9)	0.157
**Are you a day scholar or boarding student**			
Day scholar	4(5.7)	6(13.0)	
Boarding	66(94.3)	40(87.0)	0.191 ^*i*^
**Have you ever felt excluded because of your HI?**			
No	19(27.1)	18(39.1)	
Yes	51(72.9)	28(60.9)	0.175
**Reason for loss of hearing**			
Don’t Know	58(82.9)	34(73.9)	
Illnesses and medications	12(17.1)	12(26.1)	0.240
**Ever had misunderstanding about your hearing impairment from healthcare providers?**			
Yes	29(41.4)	22(48.9)	
No	18(25.7)	10(22.2)	
Not sure/ Unknown	23(32.9)	13(28.9)	0.734
**Ever experienced bullying at school due to your hearing impairment?**			
Yes	16(21.0)	8(17.4)	
No	52(76.5)	38(82.6)	0.430
**Ever experienced physical abuse at school due to your hearing impairment?**			
Yes	24(34.8)	18(39.1)	
No	45(65.2)	28(60.9)	0.635
**Ever experienced sexual abuse or harassment at school?**			
No	68(97.4)	45(97.8)	
Yes	2(2.9)	1(2.2)	1.000 ^***i***^
**Have you used drugs and/or alcohol before**			
No	64(91.4)	43(95.6)	
Yes	6(8.6)	2(4.4)	0.396
**Experienced discrimination in your community due to your hearing impairment**			
Yes	43(62.3)	24(53.3)	
No	26(37.7)	21(46.7)	0.341
**Have people treated you differently or unfairly because of your hearing impairment**			
Yes	32(45.7)	22(47.8)	
No	38(54.3)	24(52.2)	0.823
**Have you had trouble communicating with your family because of your hearing impairment**			
No	13(18.6)	9(19.6)	
Yes	57(81.4)	37(80.4)	0.894
**Hearing condition of your family members?**			
All hearing	52(75.4)	37(80.4)	
Some family members are hearing	17(24.6)	9(19.6)	0.524
**Socio-economic status**			
Low	33(47.14)	16(34.78)	
Middle	18(25.71)	19(41.30)	
High	19(27.14)	11(23.91)	0.199

*Notes 1: P-values marked with* ^i^ *were calculated using Fisher’s exact test due to low expected cell counts (<5); all others used Pearson’s chi-square test. The significance values (p < 0.05) are in bold.*

Multivariate analysis of factors associated with depression among school-going adolescents with HI revealed significant associations with living arrangements and exposure to excessive disciplinary measures. Adolescents who lived with their father only had twice the risk of depression compared to those living with both parents (IRR = 2.11, 95% CI: 1.605–2.765, p < 0.001). Those who had not witnessed excessive disciplinary measures had a significantly lower risk of depression (IRR = 0.65, 95% CI: 0.490–0.867, p = 0.003). (See Table 3).

**Table 3:** Multivariate analysis of factors associated with depression among school-going adolescents with hearing impairment (N = 116).

Variable	IRR	Robust Std. Err.	P > z	[95% Conf.
**Sex**				
Male	Reference			
Female	1.05	0.159	0.747	0.780 – 1.412
**Age group**				
10-13 years	Reference			
14-17 years	0.78	0.113	0.092	0.589 – 1.041
**Who do you stay with?**				
Both parents	Reference			
Mother only	1.28	0.222	0.147	0.915 – 1.804
Father only	2.11	0.292	0.000*	1.605 – 2.765
Others	1.34	0.342	0.246	0.816 – 2.215
**Have you experienced or witnessed excessive disciplinary measures at school?**				
Yes	Reference			
No	0.65	0.095	0.003*	0.490 – 0.867

*The significance values (p < 0.05) are in bold with an asterisk. Multivariate analysis was performed using Poisson regression with robust standard errors.*

## Discussion

This study set out to determine the prevalence of depression and associated factors among school going adolescents with hearing impairment in Kampala district, Uganda. Using lifetime prevalence of depression as the primary analytic outcome, the study found a high lifetime prevalence of depression of 60.3% among school-going adolescents with HI. The point prevalence of depression was 34.5%, indicating that over one third of the participants were experiencing depression at the time of assessment.

The reported high prevalence rates reported in this study are almost similar to an earlier study performed in South Kenya that reported a prevalence of severe depression of 54.7% [8]. This could be due to shared socio-cultural factors, including stigma, social isolation and limited mental health support for adolescents with HI as evidenced in both studies. The prevalence figures in our studies were also high when compared to the global adolescent point prevalence of depression, which was estimated at 8% and the cumulative prevalence at 19% [20]. That discrepancy shows the significant mental health burden experienced by adolescents with HI and suggests that HI compounds psychological vulnerabilities that may already be prevalent in adolescence. Another possible explanation is lack of hearing aids (79.3%), as their use has been shown to reduce the likelihood of depression among individuals with HI [21].

A study by Kinyanda et al. (2013) found that among Ugandan school children without HI, the prevalence of depression was 7.6%, which is considerably lower than the rates observed among the study demographic with HI in our study. This difference suggests that HI exacerbates mental health challenges, likely due to communication barriers, social isolation, and stigma [22]. Similar patterns have been observed in other low-resource settings where HI is associated with increased psychosocial distress and a lack of mental health support structures [23].

The point prevalence of 34.5% which is over one third of the sampled adolescents, suggests a significant current burden of depression. When considered alongside the higher lifetime prevalence observed in this study, this finding may indicate that many adolescents with HI experience recurrent episodes of depression or prolonged periods of psychological distress. The findings are consistent with Tharpar et al. [24], who highlighted that adolescent depression often follows a chronic and relapsing course, particularly in individuals with additional stressors such as disability, socio-economic hardship, or lack of support.

At multivariate analysis, the study revealed two statistically significant factors associated with an increased risk of depression among adolescents with HI; living arrangements and exposure to excessive disciplinary measures.

Adolescents residing in father-only households registered a significantly elevated risk of depression, compared to those living with both parents. This suggests that single-parent households, most notably those headed solely by fathers may pose an increased risk for depression. This could potentially be due to diminished emotional support or relational dynamics specific to this family structure [25-27]. Wang Jingxuan [28] noted that adolescents who live in single-parent households tend to develop depression more than teenagers raised in two-parent families. In the same light, Rostami demonstrated that adolescents with HI in Iran lacking stable family support were more vulnerable to mental disorders including major depression [29]. Barrett and Turner (2005) argue that single-parent households often face resource constraints, both emotional and material, that impair their capacity to buffer stressors, especially for adolescents with disabilities [30]. In the context of adolescents with HI, the lack of a second caregiver may reduce opportunities for adaptive coping strategies, such as shared problem-solving or emotional co-regulation, which are critical during adolescence [31]. Furthermore, cultural expectations of paternal roles, which may prioritize discipline over emotional nurturing, could amplify these effects in father-only homes, warranting further exploration in future studies [26].

Exposure to excessive disciplinary measures was found to be a significant predictor of depression. Adolescents who did not experience such measures demonstrated a lower likelihood of depression compared to those who did. Within the sample, a notable proportion reported harsh discipline, experiences of physical abuse and incidents of bullying which highlighted widespread exposure to adverse environments for adolescents with HI both at home and in school. These findings align with broader literature which suggests that punitive disciplinary actions both in home and educational settings which are often intended to enforce good behavior instead disproportionately harm vulnerable populations, including those with sensory impairments [32]. Such measures may erode self-esteem and intensify feelings of helplessness, both of which are established risk factors for depression [33].

A meta-analysis by Gershoff and Grogan-Kaylor (2016) found that physical punishment is consistently associated with adverse child behaviors including risks of anxiety, aggression and depression [34]. These findings highlight the long-term psychological consequences of punitive discipline and suggest that corporal punishments do not effectively modify behavior but may instead contribute to emotional distress and maladjustment. For adolescents with HI, this association may be magnified by their reliance on visual and tactile cues for communication, which can make verbal reprimands or physical interventions particularly disorienting or distressing [23].

Our study had several limitations that should be considered when interpreting findings. First, the prevalence estimates need to be interpreted with caution given the limited number of comparable studies examining depression among adolescents with hearing impairment in this context. Since the participating schools were purposively selected based on accessibility and enrolment of adolescents with HI, the findings may not be fully generalizable to all adolescents with HI across Uganda. In addition, though the instrument was adapted to USL, it has not undergone formal psychometric validation in this population which may affect diagnostic precision. Finally, the cross-sectional design limits ability to establish causal relationships between the factors‌‌ identified and depression.

## Conclusion

Our findings revealed a significantly high prevalence of depression among adolescents with HI, well above local and global rates. Key associated factors included father-only households and exposure to harsh disciplinary practices. With 21% reporting suicidal ideation, there is a clear and urgent need for targeted mental health interventions. Future studies involving more diverse and larger samples as well as longitudinal studies could further clarify the burden and determinants of depression among adolescents with HI.

## Supporting information

S1 Text Socio-demographic questionnaire used to collect participant characteristics and contextual. (PDF)

S2 Text Access details for the adapted Ugandan Sign Language MINI-KID video materials used during data collection. (PDF)
