# Supplementary material for: Depression and associated factors among primary school adolescents with hearing impairment in Kampala district, Uganda: A cross-sectional study
Source: PLOS Ment Health. 2026 Jun 18;3(6):e0000588. doi: 10.1371/journal.pmen.0000588 (PMC13278404; doi:10.1371/journal.pmen.0000588)
Supplement: S1 Text — (PDF) [file pmen.0000588.s001.pdf]

SOCIO-DEMOGRAPHIC QUESTIONNAIRE

Please answer all questions honestly; you will not be guided on your responses. Please feel free to ask if you need any of the questions explained to you.

The questionnaire is very brief and should take 5 to 10 minutes to complete.

Section 1: Socio-Demographic Information

1, Age: _______

2, Gender:

Male ☐

Female ☐

Other (Please specify: _______)

3, Grade/Class: _______

4, What age did you start school? _______

5, Boarding or day scholar?

Boarding ☐

Day ☐

6, At what age did you know about your hearing loss?

I was born with HI ☐

Before 5 years ☐

5 years and above ☐

7, What was the reason for your loss of hearing?

Malaria ☐

Given medicine like quinine ☐

Ear infection ☐

Noise related (was using headphones, loud music) ☐

Don't know ☐

Other cause : ___________________________________

Section 2: Health System Related Factors

8, Have you been assessed for hearing loss before?

Ye ☐

No ☐

9, Do you have any physical illness?

Yes ☐

No ☐

If yes, specify ______________________________

10, Are you currently seeing any health worker?

Yes ☐

N ☐

If yes, please specify which professionals you are seeing (e.g., doctor, speech and language therapists, audiologists) ______________________________

11, Are you currently taking any medication?

Yes ☐

No ☐

If yes, please specify the medication ______________________________

12, Are there healthcare workers at healthcare facilities where you seek treatment who know sign language?

Yes ☐

No ☐

Not sure/Unknown

13, Have you ever had misunderstanding about your hearing impairment from healthcare providers?

Yes ☐

No ☐

Not sure/Unknown ☐

If yes, please provide examples of the misunderstandings or difficulties you have faced.

Section 3: School Related Factors

14, Have you ever experienced bullying at school due to your hearing impairment?

Yes ☐

No ☐

Not sure/Unknown ☐

If yes, please describe the types of bullying you have experienced and its impact on your well-being.

15, Have you ever experienced physical abuse at school due to your hearing impairment

Yes ☐

No ☐

Not sure/Unknown ☐

If yes, please describe the incidents of physical abuse and its impact on your well-being.

16, Have you ever experienced sexual abuse or harassment at school?

Yes ☐

No ☐

Not sure/Unknown ☐

If yes, please describe the incidents of sexual abuse and its impact on your well-being.

17, Have you ever witnessed any instances of unfair or excessive discipline at your school?

Yes ☐

No ☐

Not sure/Unknow ☐

If yes, please describe these incidents and their impact on your well-being.

Section 4: Individual Factors

18, Do you wear a hearing aid?

Yes ☐

No ☐

19, Do you wear a cochlear implant?

Yes ☐

No ☐

20, Have you used drugs and/or alcohol before?

Yes ☐

No ☐

If yes, please specify the type of drug(s) used and how often.

Section 5: Community Factors

21, Have you experienced discrimination in your community due to your hearing impairment?

Yes ☐

No ☐

Not sure/Unknown ☐

If yes, please provide examples of discrimination you have encountered.

22, Have people treated you differently or unfairly because of your hearing impairment?

Yes ☐

No ☐

Not sure/Unknown ☐

If yes, please describe those instances and how they have affected you.

23, Have you ever been physically or sexually abused in the community (outside school)?

Yes ☐

No ☐

Not sure/Unknown ☐

If yes, please describe the incidents of abuse and its impact on your well-being.

Section 6: Family Factors

24, Who do you stay with?

Both parents ☐

Mother only ☐

Father only ☐

Others (specify)

If not staying with both reasons, give reason (parents died, separated…)

25, What is the hearing condition of your family members?

All hearing ☐

Some family members are hearing, some have hearing impairment ☐

All family members have hearing impairment ☐

Not sure/Unknown ☐

26, Have you had trouble communicating with your family because of your hearing impairment?"

Yes ☐

No ☐

Not sure/Unknown ☐

If yes, please describe the nature of the communication gaps and how it affects your relationships.

27, Have you ever felt excluded by your family due to your hearing impairment?

Yes ☐

No ☐

Not sure/Unknown ☐

If yes, please elaborate on the experiences of feeling excluded.

28, What is your parents' source of income?

29, Do you have any of the following in your home? (only items that work) Tick all that apply.

Mobile phone (ring / SMS)/telephone	No ☐	Yes ☐
Smartphone (internet option)	No ☐	Yes ☐
Radio	No ☐	Yes ☐
TV	No ☐	Yes ☐
Lap top/Computer	No ☐	Yes ☐

Refrigerator	No ☐	Yes ☐
Bicycle	No ☐	Yes ☐
Motor cycle/scooter	No ☐	Yes ☐
Car/truck	No ☐	Yes ☐

Section 8: Additional Comments

Please provide any additional comments or information you think might be relevant to the study:

Thank you for your participation. Your responses will be kept confidential, and your cooperation is greatly appreciated.
