# Supplementary material for: Depression and associated factors among primary school adolescents with hearing impairment in Kampala district, Uganda: A cross-sectional study
Source: PLOS Ment Health. 2026 Jun 18;3(6):e0000588. doi: 10.1371/journal.pmen.0000588 (PMC13278404; doi:10.1371/journal.pmen.0000588)
Supplement: S2 Text — (PDF) [file pmen.0000588.s002.pdf]

Adapted MINI-KID Ugandan Sign Language Videos are accessible via this google drive link:

https://drive.google.com/drive/folders/1Hks6caNkJTT-WrLC9fUaYqpHkwcmH-yV?usp=sharing
